# Parliament and the Profession

**Published:** 1918-11-02

**Authors:** 


### Parliament and the Profession.

### Artificial Limbs.

THE Minister of Pensions, Mr. Hodge, announced that the number of beds in fitting centres for artificial limbs has increased from 972 in February 1918 to 2,196 in October. It has not yet been found possible to standardise limbs, as no specimen brought to the knowledge of the Advisory Council has shown such advantages over any other as to warrant it being made the standard pattern.

### Fazakerley Hospital, Liverpool.

MR. MACPHERSON informed Mr. Pennefather that he would inquire into the suggestion that a part of Fazakerley Hospital has been used for typists or for storage purposes to the exclusion of tuberculosis cases.

### Rabies in Devon and Cornwall.

MR. PROTHERO stated that there had been thirty-five cases of rabies in Devon and five in Cornwall confirmed since September 7. He had no knowledge of the truth of a statement that the outbreak had been caused by a dog brought in an aeroplane from France, but was aware of the possibility of contagion being spread in this way, and both the Admiralty and the Royal Flying Corps had given every assistance by warning officers of the serious breach of regulations that might in this way be involved.

### Cooking in London Military Hospitals.

MR. MACPHERSON informed Mr. Gilbert that some complaints as to the way food is cooked in the London military hospitals have been received, and an officer from the War Office has been sent to investigate the matter and make suggestions with a view to improvement. The whole question of cooking in military hospitals is receiving close attention, and the appointment of expert lady cooks to act as inspectors of cooking in military hospitals is at present under consideration.

